# Fetal Pancreatic Circumference as a Predictor of Gestational Diabetes Mellitus During 75 g OGTT

**DOI:** 10.3390/jcm14155414

**Published:** 2025-08-01

**Authors:** Mehmet Can Keven, Ece Aydoğdu, Banu Derim Yeğen, Ebru Yucel, Zafer Bütün, Atakan Tanaçan

**Affiliations:** 1 Department of Gynecology and Obstetrics, Division of Perinatology, Eskişehir City Hospital, Eskişehir 26080, Turkey; 2 Department of Gynecology and Obstetrics, Eskişehir City Hospital, Eskişehir 26080, Turkey; ece-aydogdu@hotmail.com (E.A.); drderimbanu@outlook.com (B.D.Y.); 3 Department of Adult Medicine, ChristianaCare Hospital, Wilmington, DE 19801, USA; ebru.yucel@christianacare.org; 4 Private Perinatology Clinic, Eskişehir 26090, Turkey; zaferbutun@hotmail.com; 5 Department of Gynecology and Obstetrics, Division of Perinatology, Ankara Bilkent City Hospital, Ankara 06800, Turkey; atakantanacan@yahoo.com

**Keywords:** fetal pancreas circumference, fetal pancreas circumference percentile, 75 g oral glucose tolerance test, gestational diabetes mellitus

## Abstract

**Objectives:** The objective of this study was to investigate the relationship between the simultaneous 75 g Oral glucose tolerance test (OGTT), gestational diabetes mellitus (GDM), and fetal pancreatic circumference at 24–28 weeks of gestation. **Methods:** This prospective case–control study was conducted between September 2024 and February 2025 at our perinatology clinic, which provides tertiary health care services. The correlation between the 75 g OGTT, GDM, and pancreatic circumference was assessed by comparing fetal pancreatic circumference between the groups with and without GDM at the time of diagnosis. **Results:** A total of 130 pregnant patients were recruited for this` study, with 64 patients forming the GDM group and 66 patients forming the control group. Fetal pancreas circumference (7.0 cm vs. 6.4 cm, *p* < 0.001), fetal pancreas circumference percentile (88.5 vs. 52, *p* < 0.001), and the rate of fetal pancreas size >90th percentile (15.6% vs. 3%, *p* < 0.001) were significantly higher in the GDM group compared to the control group. **Conclusions:** Although our findings demonstrate a statistically significant correlation between fetal pancreatic circumference and GDM, diagnostic performance remains modest. Therefore, fetal pancreatic circumference should be interpreted as a supportive marker, such as family history, rather than a definitive marker for identifying individuals at risk for GDM.

## 1. Introduction

Gestational diabetes mellitus (GDM) is a condition characterized by glucose intolerance first diagnosed during pregnancy. It affects approximately 15% of pregnant individuals, with its incidence rising globally due to factors such as increasing maternal age, the growing prevalence of obesity, and evolving screening strategies and diagnostic criteria [1].

In 1952, Jørgen Pedersen proposed that maternal hyperglycemia leads to increased transplacental glucose transfer, which stimulates excessive insulin production by the fetal pancreatic β-cells. This process promotes fetal overgrowth and the development of macrosomia [2]. Indeed, the Pedersen hypothesis has been foundational in elucidating the maternal, fetal, and neonatal pathophysiological consequences of gestational diabetes mellitus (GDM) [3]. Fetal pancreatic β-cell hypertrophy and hyperinsulinism result in increased fat deposition, contributing to intrauterine overgrowth, neonatal hypoglycemia, and a heightened risk of obesity and type 2 diabetes in later life [3,4]. In GDM, maternal metabolic status affects maternal structural morphology as well as fetal metabolic status and subsequent fetal structural morphology [3,4,5].

Ultrasonographic findings such as fetal macrosomia and polyhydramnios in gestational diabetes mellitus (GDM) have long been recognized and extensively studied [6]. However, research on the ultrasonographic evaluation of the fetal pancreas remains limited and has only recently gained attention. In 2017, Kivilevitch et al. introduced a fetal pancreas measurement technique and established a reference range for normal fetal pancreatic circumference throughout pregnancy [7]. More recently, in 2021, Gilboa et al. demonstrated a possible correlation between glycemic control, pancreatic size, and gestational age in both pregestational and gestational diabetes [8].

Malhotra et al. (2022) [9] reported that all fetal biometric parameters, including pancreatic measurements, were increased in cases of GDM compared to non-GDM cases at the same gestational age in a study analyzing abortion specimens. Additionally, histological examination of the fetal pancreas revealed islet of Langerhans hyperplasia in abortion cases with GDM [9].

The primary aim of our study was to investigate the correlation between fetal pancreatic circumference and the 75 g oral glucose tolerance test (OGTT) results at 24–28 weeks of gestation during GDM diagnosis. Additionally, we aimed to review current screening, diagnostic, and treatment protocols in light of these findings.

## 2. Materials and Methods

This prospective case–control study was conducted at the Perinatology Clinic of the Republic of Turkey Ministry of Health Eskişehir City Hospital, a tertiary care center, between September 2024 and February 2025.

Inclusion criteria were as follows: (1) singleton pregnancy; (2) absence of fetal or placental anomalies; (3) no known pregestational diabetes mellitus; (4) gestational age between 24 and 28 weeks based on the last menstrual period and confirmed by first-trimester crown-rump length measurement; and (5) low-risk medical and obstetric profile.

Exclusion criteria included the following: (1) multiple pregnancies; (2) fetal chromosomal or structural abnormalities; (3) technical limitations in fetal pancreatic imaging, such as maternal morbid obesity; (4) maternal or fetal high-risk conditions (including preeclampsia, chronic hypertension, thyroid disorders, preterm labor, autoimmune disease, or pregestational diabetes); and (5) diagnosis of GDM using a two-step screening approach. The latter was excluded to ensure methodological consistency and group homogeneity.

In accordance with institutional practice and international guidelines, including those from FIGO (2015), WHO (2013), and IADPSG (2010) [10,11,12], all pregnant individuals were screened for GDM using the one-step 75 g OGTT between 24 and 28 weeks of gestation. The diagnostic thresholds for normal values were defined as fasting plasma glucose <92 mg/dL, 1 h glucose <180 mg/dL, and 2 h glucose <153 mg/dL. A single elevated value was considered sufficient for the diagnosis of GDM [10,11,12].

Pregnant patients who tested positive for GDM on the 75 g OGTT performed between 24 and 28 weeks of gestation and who met the inclusion and exclusion criteria were classified as having GDM. Maternal characteristics—including age, gravidity, parity, body mass index (BMI), weight gain, gestational age, smoking status, history of polycystic ovary syndrome (PCOS), and family history of diabetes mellitus—were recorded. Additionally, blood HbA1c levels and fetal ultrasonographic evaluations were performed concurrently with the 75 g OGTT in all participants.

### 2.1. Ultrasonographic Evaluation

Ultrasound assessments, including fetal pancreatic circumference measurement, were performed between 24 and 28 weeks of gestation, at the same visit as the 75 g oral glucose tolerance test. All participants first underwent a general fetal anatomical assessment, followed by the estimation of fetal weight using the Hadlock formula, which incorporates biparietal diameter, head circumference, fetal abdominal circumference, and femur length. For pancreatic imaging, fetal pancreatic circumference measurements and percentile determinations were conducted following the methodology described by Kivilevitch et al. (2016) [7].

In our practice, the most suitable abdominal imaging plane was a transverse view at the level of the stomach and liver. However, as described by Güleroğlu et al., a slightly oblique plane with the fetal spine positioned between 3 and 5 o’clock or between 7 and 9 o’clock provided satisfactory visualization [13]. The stomach played a key role in identifying the pancreas; by tilting the transducer posterior to the stomach to locate the pancreatic tail, the entire pancreas appeared as a continuous longitudinal structure when the probe was rotated according to the fetal back position. The pancreas was visualized as a slightly echogenic structure located posterior to the stomach, anterior to the left kidney, aorta, superior vena cava, and vertebral column, extending toward the gallbladder on the right.

**Figure 1:**
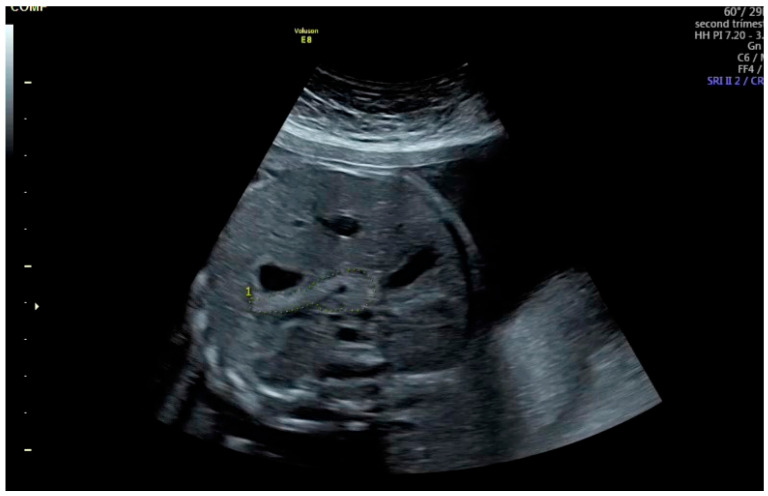
Gray scale ultrasound showing fetal pancreas circumference with dotted lines. The pancreas is seen as a slightly echogenic structure behind the stomach, anterior to the left kidney, aorta, vena cava superior and vertebral spine, and extending to the gallbladder on the right.

After freezing the image, the pancreatic circumference was measured using the freehand trace method (Figure 1). The measurement was repeated three times, and the mean value was recorded. Fetal pancreatic circumference percentiles were determined according to the reference data established by Kivilevitch et al. [7,14,15].

A control group was formed by performing the same evaluations in pregnant individuals who met the inclusion and exclusion criteria and tested negative for GDM on the 75 g OGTT performed between 24 and 28 weeks of gestation. Control patients were randomly selected from the eligible population to ensure unbiased group formation. Patients who were unable to tolerate the OGTT—such as those who experienced significant adverse effects during the test—or who were receiving hypoglycemic medications (e.g., metformin or insulin) at the time of testing were excluded from both groups to ensure untreated metabolic status during concurrent ultrasound and OGTT evaluation. Demographic characteristics, clinical features, fetal pancreatic circumference measurements, and fetal pancreatic circumference percentiles were compared between the GDM group and the control group. This comparison aimed to evaluate the correlation between maternal glycemic status, as determined by the 75 g OGTT—the standard screening and diagnostic test for GDM—and fetal pancreatic size measured concurrently.

Written informed consent was obtained from all participants. Fetal ultrasonographic evaluations were performed by a single highly experienced maternal–fetal specialist (MCK) using a Voluson E8 ultrasound system (GE Medical Systems) with standard transabdominal curvilinear transducer equipment.

This study was conducted in accordance with the Declaration of Helsinki (revised 2013). The study protocol was approved by the Ethics Committee of the Republic of Turkey Ministry of Health, Eskişehir City Hospital (approval date: 19 September 2024; decision number: ESH/BAEK 2024/59).

### 2.2. Statistical Analysis

Data were analyzed using IBM SPSS Statistics (Version 22). The Shapiro–Wilk test and visual histograms were used to assess the normality of data distribution. As the data were not normally distributed, continuous variables are presented as medians with interquartile ranges (IQR), while categorical variables are presented as counts and percentages. The Mann–Whitney U test was used to compare median values between groups, and the chi-square test was applied to compare categorical variables. A receiver operating characteristic (ROC) analysis was performed to evaluate the performance of fetal pancreatic circumference and fetal pancreatic circumference percentile in predicting GDM development, with the Youden index employed to determine the optimal cut-off values for maximum sensitivity and specificity. Correlation analysis was conducted to assess the relationships between fetal pancreatic circumference, fetal pancreatic circumference percentile, 75 g OGTT results, and HbA1c values. Finally, a univariate analysis was performed to identify independent risk factors for GDM. Additionally, a multivariate logistic regression analysis was conducted to determine independent predictors of GDM, adjusting for relevant maternal and fetal parameters. A *p*-value of <0.05 was considered statistically significant.

## 3. Results

A total of 130 pregnant patients were recruited for the study, with 64 patients constituting the GDM group and 66 patients forming the control group. The demographic characteristics and clinical features of the study population are summarized in Table 1. Statistically significant differences were observed between the GDM and control groups in maternal age, fetal pancreatic circumference, fetal pancreatic circumference percentile, the rate of fetal pancreatic circumference >90th percentile, HbA1c levels, fasting plasma glucose, 75 g OGTT 1st-hour and 2nd-hour glucose levels, and family history of diabetes mellitus (*p* < 0.05).

Fetal pancreatic circumference (7.0 vs. 6.4 cm, *p* < 0.001), fetal pancreatic circumference percentile (88.5 vs. 52, *p* < 0.001), and the rate of fetal pancreatic circumference > 90th percentile (15.6% vs. 3%, *p* < 0.001) were significantly higher in the GDM group compared to the control group.

Table 2 illustrates the role of fetal pancreatic circumference and fetal pancreatic circumference percentile in predicting GDM. The optimal cut-off value for fetal pancreatic circumference was 6.8 cm (sensitivity: 70.3%, specificity: 60.6%, AUC: 0.68, 95% CI: 0.59–0.77, *p* < 0.001). For fetal pancreatic circumference percentile, the optimal cut-off value was 73.5th percentile (sensitivity: 70%, specificity: 86.4%, AUC: 0.83, 95% CI: 0.76–0.90, *p* < 0.001).

**Figure 2:**
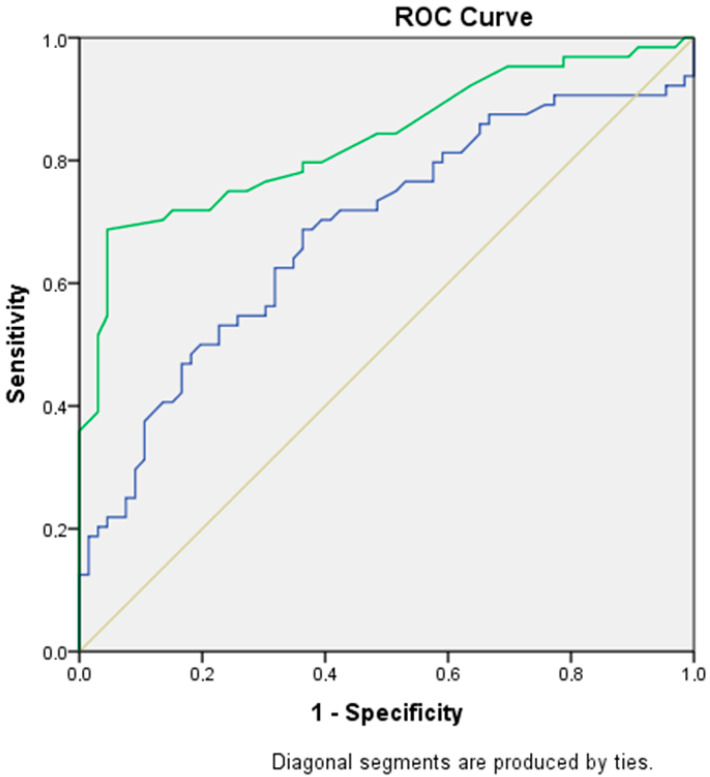
ROC curve for predicting GDM (gestational diabetes mellitus) via fetal pancreas circumference and fetal pancreas circumference percentile. (green line: fetal pancreas circumference percentile, blue line: fetal pancreas circumference).

Figure 2 presents the ROC curve for predicting GDM based on fetal pancreatic circumference and fetal pancreatic circumference percentile.

Table 3 presents the correlation analysis between fetal pancreatic circumference, fetal pancreatic circumference percentile, and fasting plasma glucose, 75 g OGTT 1st-hour glucose, 75 g OGTT 2nd-hour glucose, and HbA1c. A statistically significant positive but weak correlation was observed between fetal pancreatic circumference and both fasting plasma glucose and 75 g OGTT values. In contrast, a statistically significant, positive, and moderate to strong correlation was found between fetal pancreatic circumference and HbA1c levels. Additionally, a statistically significant positive, weak to moderate correlation was observed between fetal pancreatic circumference percentile and fasting plasma glucose, 75 g OGTT values, and HbA1c levels.

Table 4 presents the results of the univariate regression analysis for identifying independent risk factors for the development of GDM. The analysis revealed that a fetal pancreatic circumference percentile >90th percentile (OR: 5.9, 95% CI: 1.2–28.2, *p* = 0.02), fasting plasma glucose ≥92 mg/dL (OR: 3.5, 95% CI: 1.7–15.3, *p* < 0.001), HbA1c ≥5.7 (OR: 5.1, 95% CI: 1.6–16.4, *p* = 0.05), and a family history of diabetes mellitus (OR: 2.4, 95% CI: 1.1–4.9, *p* = 0.01) were significantly associated with an increased risk of developing GDM.

Table 5 presents the independent predictors of gestational diabetes mellitus (GDM), as identified through a multivariate logistic regression model that included maternal age, BMI, family history of diabetes, HbA1c, fetal pancreatic percentile, and absolute pancreatic circumference. The analysis revealed that both HbA1c (β = −3.02, *p* = 0.0004) and fetal pancreatic percentile (β = −0.08, *p* < 0.001) were statistically significant predictors of GDM. Absolute pancreatic circumference demonstrated a borderline association (β = 0.73, *p* = 0.072), while maternal age, BMI, and family history of diabetes did not reach statistical significance (*p* > 0.05). These findings highlight the potential utility of combining metabolic indicators (HbA1c) with fetal ultrasonographic parameters in the assessment of GDM risk.

**Figure 3:**
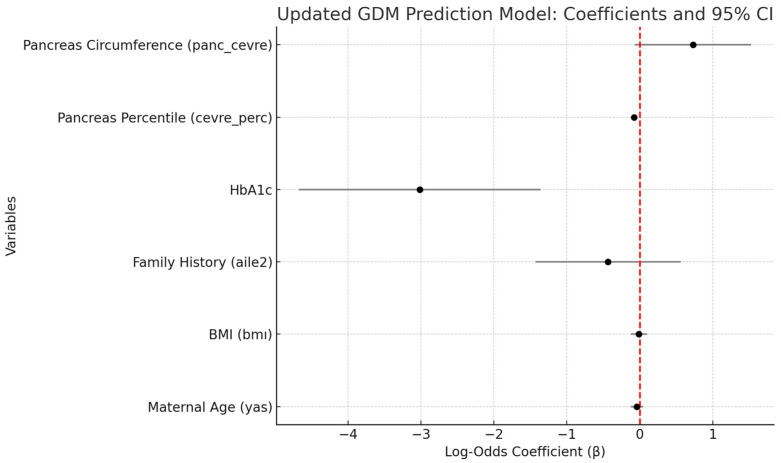
Forest plot of model coefficients.

Figure 3 presents a scatter plot illustrating the correlation between fetal pancreatic circumference and 2 h plasma glucose levels obtained during the 75 g OGTT. A statistically significant positive correlation was observed (Spearman’s ρ = 0.42, *p* < 0.001), indicating that higher 2 h OGTT glucose levels were associated with increased fetal pancreatic circumference.

## 4. Discussion

According to the Pedersen hypothesis, in GDM, the primary expectation is that maternal hyperglycemia and associated fetal hyperglycemia lead to increased pancreatic activity, insulin production, and pancreatic growth [16]. This has driven investigations into the effects of GDM on pancreatic morphology. In our study, we evaluated fetal pancreatic circumference and pancreatic circumference percentile using ultrasonography in GDM cases diagnosed with a positive 75 g OGTT at 24–28 weeks of gestation. Our study had two main objectives: (1) to determine whether there was evidence of fetal pancreatic involvement at the time of GDM diagnosis, which could suggest that this involvement preceded the diagnosis, and (2) to explore the positive correlation between ultrasonographic pancreatic circumference, fetal pancreatic circumference percentile, and the 75 g OGTT, which is used in GDM screening and diagnosis. This correlation could potentially support the use of ultrasonographic pancreatic size assessment to identify populations at high risk for GDM. Indeed, our findings revealed statistically significant increases in both pancreatic circumference and fetal pancreatic circumference percentile in the GDM group compared to the control group based on simultaneous ultrasonographic evaluations performed during the diagnosis of GDM at 24–28 weeks.

In our study, optimal cut-off values for fetal pancreatic circumference and fetal pancreatic circumference percentile were identified for the prediction of GDM. Based on these findings, elevated fetal pancreatic circumference and percentile values observed during routine ultrasound may serve as supportive markers of underlying gestational diabetes mellitus in pregnancies between 24 and 28 weeks and could reinforce clinical suspicion in borderline or equivocal cases.

GDM is a metabolic disorder that affects a significant proportion of pregnancies, and if not diagnosed and managed early, it can lead to severe maternal, fetal, and neonatal complications [3,17]. These complications include both short-term and long-term outcomes such as preeclampsia, fetal macrosomia, stillbirth, neonatal hypoglycemia, neonatal electrolyte imbalances, neonatal hyperbilirubinemia, shoulder dystocia, operative delivery, fetal–maternal birth trauma, respiratory distress syndrome, maternal and neonatal obesity, type 2 diabetes, cardiovascular diseases, and other metabolic disorders [3,4,6,17,18,19,20]. However, with early diagnosis, dietary modifications, exercise, and pharmacological treatment (when necessary), normal blood glucose levels can be achieved, significantly reducing maternal, fetal, and neonatal complications [3,4,18,21].

Insulin resistance increases markedly in pregnant individuals after 24 weeks, and when pancreatic insulin secretion is insufficient to maintain euglycemia, hyperglycemia develops [17,18]. As a result, screening for GDM is recommended during this period, using either a one-step or two-step approach. The FIGO 2015, WHO 2013, and IADPSG 2010 guidelines recommend universal screening using a two-hour fasting 75 g OGTT, with a one-step testing approach preferred over the two-step method for GDM screening and diagnosis [10,11,12]. This approach facilitates screening with a direct diagnostic test, making the 75 g OGTT the preferred method in health institutions serving large patient populations. Given that our hospital serves a large population, we used the 75 g OGTT, as described in the materials and methods, because it offers the advantage of being a direct diagnostic test for GDM. Differences in the methodologies used for GDM screening and diagnosis across studies can complicate the comparison of results.

In a study conducted by Gilboa et al. in 2024 [14], fetal pancreatic size was measured before GDM screening at 20–25 weeks in low-risk pregnant patients. The authors concluded that early assessment of fetal pancreatic size could contribute to earlier diagnosis and treatment of GDM, potentially reducing fetal exposure to high glucose levels [14]. However, their study had several methodological differences compared to ours, including its retrospective design, a smaller cohort size, the use of a two-step approach for GDM diagnosis (unlike our one-step approach), and an earlier timing of fetal ultrasonographic evaluation (20–25 weeks vs. 24–28 weeks). Despite these differences, our findings are consistent and support each other, highlighting the potential role of fetal pancreatic size in the early detection of GDM.

Conversely, some studies have found no significant association between fetal pancreatic size and GDM. For instance, Güleroğlu et al. (2023) reported that neither fetal pancreatic measurements nor maternal biomarkers—such as glycated albumin and insulin-regulated aminopeptidase—were effective in the early prediction of GDM [13]. These discrepant findings may be explained by variations in ultrasound timing (early second trimester and 24–28 weeks vs. solely at 24–28 weeks), differences in sample size of GDM cases (19 vs. 64), patient risk profiles, and inconsistencies in measurement techniques.

In addition to univariate analysis, we conducted a multivariate logistic regression to identify independent predictors of GDM. In the adjusted model, HbA1c and fetal pancreatic circumference percentile emerged as strong and statistically significant predictors, while absolute pancreatic circumference showed a borderline association. These findings suggest that fetal pancreatic measurements—particularly percentile values—retain predictive value even after controlling for maternal metabolic and demographic factors. Notably, maternal age, BMI, and family history of diabetes did not remain significant after adjustment, indicating that fetal ultrasonographic parameters may contribute to the diagnostic insight during OGTT screening.

The strengths of our study include its prospective case–control design, the fact that fetal ultrasonographic evaluations were performed by a single experienced maternal–fetal specialist, a relatively large patient cohort, and the simultaneous performance of both 75 g OGTT and fetal ultrasonographic evaluations.

This study has several limitations. The patient population consisted of selected cases, and individuals in whom fetal pancreatic imaging could not be performed, due to technical challenges such as maternal obesity, were excluded. Additionally, intra-observer and inter-observer variability analyses were not conducted for the fetal pancreatic measurements. Although all ultrasonographic evaluations were performed by a single experienced examiner to ensure consistency, the reproducibility of the measurements was not objectively assessed.

Our findings suggest that fetal pancreatic involvement may be present at the time of GDM diagnosis; however, the cross-sectional design of the study limits the ability to establish causality or determine temporal relationships. Untreated high glucose exposure from the onset of fetal pancreatic involvement until the diagnosis of GDM could contribute to the persistence of GDM-related complications during the fetal, neonatal, and adult stages, despite treatment following diagnosis. The potential impact of fetal pancreatic involvement on the development of metabolic disorders in adulthood presents an intriguing area for further investigation.

Furthermore, the statistically significant positive correlation between fetal pancreatic circumference and the 75 g OGTT results suggests that this measurement functions more as a concurrent diagnostic marker than a predictive one. Our findings indicate that fetal pancreatic circumference measured between 24 and 28 weeks is a strong concurrent marker for GDM; however, the primary objective remains the identification of earlier predictive markers. Various first-trimester indicators have been investigated in this context. For example, Sirico et al. demonstrated that pregestational diabetes can alter fetal heart rate in the first trimester, potentially serving as an early predictor of subsequent GDM development [22,23]. Future studies assessing the predictive value of fetal pancreatic size should adopt a longitudinal design and focus on the first or early second trimester.

The biological mechanism linking maternal hyperglycemia to fetal pancreatic growth likely involves fetal hyperinsulinemia and insulin-mediated organomegaly. Histological evidence, such as the study by Malhotra et al. (2022), has demonstrated islet cell hyperplasia in fetal pancreatic tissue from GDM pregnancies that ended in abortion, supporting this hypothesis [9]. Additionally, recent placental studies suggest that insulin signaling defects and inflammatory changes associated with GDM may contribute to fetal pancreatic remodeling [24]. These findings underscore the need for further research into the histopathological correlates of ultrasonographic observations in GDM.

## 5. Conclusions

Although our study demonstrates a statistically significant correlation between fetal pancreatic circumference and GDM, the diagnostic performance of this marker remains modest. Therefore, fetal pancreatic circumference should be regarded as a supportive indicator—similar to family history—rather than as a standalone marker for identifying individuals at risk for GDM. Our study specifically aimed to investigate whether fetal pancreatic circumference, measured concurrently with routine GDM screening at 24–28 weeks, correlates with maternal glucose levels and GDM status. We do not propose pancreatic measurement as an independent screening tool but rather as a potential marker reflecting concurrent maternal metabolic status at the time of diagnosis. The significant correlation we observed suggests that fetal pancreatic size may mirror maternal glycemic conditions, offering insights into fetal metabolic adaptation in GDM. Future research should explore whether fetal pancreatic measurements performed earlier in pregnancy—such as during the routine 20-week anatomy scan—could have predictive value for identifying pregnancies at increased risk of developing GDM.

## Figures and Tables

**Table 1:** Comparison of demographic characteristics and clinical features between the study and the control groups.

Variables	GDM Group (n = 64)	Control Group (n = 66)	*p*-Value
Maternal age (years) (median, IQR)	31.5 (11)	28 (8)	**0.04**
Gravidity (median, IQR)	2 (2)	2 (1.25)	0.44
Parity (median, IQR)	1 (1)	1 (1.25)	0.58
BMI (kg/m^2^)	29 (6.5)	27.7 (4)	0.06
Weight gain (kg) (median, IQR)	7 (5)	9 (6)	0.16
Gestational age (weeks) (median, IQR)	26 (2.75)	26 (3)	0.58
EFW percentile (median, IQR)	48.5 (7.5)	40 (10.5)	0.17
Fetal pancreas circumference (cm) (median, IQR)	7.0 (1)	6.4 (1)	**<0.001**
Fetal pancreas circumference percentile (median, IQR)	88.5 (30.2)	52 (18.5)	**<0.001**
Fetal pancreas >90th percentile rate (n, %)	10 (15.6%)	2 (3%)	**0.01**
HbA1c (median, IQR)	5.4 (0.5)	5.1 (0.4)	**<0.001**
Fasting plasma glucose (mg/dL) <0.001	92.5 (14.5)	81 (10.5)	**<0.001**
75 g OGTT 1st hour (mg/dL) <0.001	191 (37.2)	126.5 (44)	**<0.001**
75 g OGTT 2nd hour (mg/dL) <0.001	139.5 (41.2)	104.5 (29)	**<0.001**
Fasting plasma glucose ≥92 mg/dL rate (n, %)	34 (53%)	0 (0%)	**<0.001**
75 g OGTT 1st hour ≥180 mg/dL rate (n, %)	43 (67.2%)	0 (0%)	**<0.001**
75 g OGTT 2nd hour ≥153 mg/dL rate (n, %)	26 (40.6%)	0 (0%)	**<0.001**
Smoking rate (n, %)	10 (15.6%)	13 (19.7%)	0.54
PCOS rate (n, %)	4 (6.3%)	3 (4.5%)	0.66
Family history of diabetes mellitus rate (n, %)	34 (53.1%)	21 (31.8%)	**0.01**

GDM: Gestational diabetes mellitus, IQR: Interquartile range, BMI: Body mass index, EFW: Estimated fetal weight, OGTT: Oral glucose tolerance test, PCOS: Polycystic ovary syndrome.

**Table 2:** ROC analysis for the performance of fetal pancreas circumference and fetal pancreas circumference percentile for the prediction of GDM.

	Cut-Off Value	Sensitivity	Specificity	AUC	95% CI	*p*-Value
Fetal pancreas circumference (cm)	6.8	70.3%	60.6%	0.68	0.59–0.77	**<0.001**
Fetal pancreas circumference percentile	73.5	70%	86.4%	0.83	0.76–0.90	**<0.001**

AUC: Area under the curve, CI: Confidence interval.

**Table 3:** Correlation analysis between fetal pancreas circumference, fetal pancreas circumference percentile, HbA1c, fasting plasma glucose, 75 g OGTT 1st hour, and 75 g OGTT 2nd hour values.

	Fetal Pancreas Circumference		Fetal Pancreas Circumference Percentile	
	**r**	***p*-Value**	**r**	***p*-Value**
Fasting plasma glucose (mg/dL)	0.20	**0.02**	0.28	**0.001**
75 g OGTT 1st hour (mg/dL)	0.28	**0.001**	0.49	**<0.001**
75 g OGTT 2nd hour (mg/dL)	0.22	**0.01**	0.42	**<0.001**
HbA1c	0.60	**<0.001**	0.23	**0.008**

OGTT: Oral glucose tolerance test.

**Table 4:** Univariate regression analysis for determining the independent risk factors for GDM development.

Variables	OR	95% CI	*p*-Value
Fetal pancreas circumference percentile >90th	5.9	1.2–28.2	**0.02**
Fasting plasma glucose ≥92 mg/dL	3.5	1.7–15.3	**<0.001**
HbA1c ≥5.7	5.1	1.6–16.4	**0.005**
Family history for diabetes mellitus	2.4	1.1–4.9	0.01

GDM: Gestational diabetes mellitus, OR: Odds ratio, CI: confidence interval.

**Table 5:** Logistic regression results (updated model).

Variable	Coefficient (β)	Std. Error	Z-Value	*p*-Value	95% CI Lower	95% CI Upper
Intercept	17.6654	4.5035	3.9226	0.0001	8.8388	26.4921
Maternal Age (yas)	−0.0395	0.0427	−0.9245	0.3552	−0.1232	0.0442
BMI (bmı)	−0.0123	0.0561	−0.2184	0.8271	−0.1223	0.0978
Family History (aile2)	−0.4351	0.5073	−0.8577	0.391	−1.4294	0.5592
HbA1c	−3.0155	0.8448	−3.5696	0.0004	−4.6711	−1.3598
Pancreas Percentile (cevre_perc)	−0.0796	0.0161	−4.9311	0.0	−0.1112	−0.0479
Pancreas Circumference (panc_cevre)	0.7311	0.4067	1.7977	0.0722	−0.066	1.5281

## Data Availability

The data that support the findings of this study are available from the corresponding author upon reasonable request.
